# Insight into metabolic pathways of the potential biofuel producer, *Paenibacillus polymyxa* ICGEB2008

**DOI:** 10.1186/s13068-015-0338-4

**Published:** 2015-09-25

**Authors:** Nidhi Adlakha, Thomas Pfau, Oliver Ebenhöh, Syed Shams Yazdani

**Affiliations:** Synthetic Biology and Biofuels Group, International Centre for Genetic Engineering and Biotechnology, Aruna Asaf Ali Marg, New Delhi, India; Institute of Complex Systems and Mathematical Biology, University of Aberdeen, Aberdeen, UK; Life Sciences Research Unit, University of Luxembourg, Luxembourg City, Luxembourg; Cluster of Excellence on Plant Sciences (CEPLAS), Heinrich-Heine-University, Universitätsstraße 1, 40225 Düsseldorf, Germany

**Keywords:** *Paenibacillus polymyxa*, Metabolic modeling, Ethanol, 2,3-Butanediol, Metabolic engineering, Redox metabolism

## Abstract

**Background:**

*Paenibacillus polymyxa* is a facultative anaerobe known for production of hydrolytic enzymes and various important biofuel molecules. Despite its wide industrial use and the availability of its genome sequence, very little is known about metabolic pathways operative in the Paenibacillus system. Here, we report metabolic insights of an insect gut symbiont, *Paenibacillus polymyxa* ICGEB2008, and reveal pathways playing an important role in the production of 2,3-butanediol and ethanol.

**Result:**

We developed a metabolic network model of *P. polymyxa* ICGEB2008 with 133 metabolites and 158 reactions. Flux balance analysis was employed to investigate the importance of redox balance in ICGEB2008. This led to the detection of the Bifid shunt, a pathway previously not described in Paenibacillus, which can uncouple the production of ATP from the generation of reducing equivalents. Using a combined experimental and modeling approach, we further studied pathways involved in 2,3-butanediol and ethanol production and also demonstrated the production of hydrogen by the organism. We could further show that the nitrogen source is critical for metabolite production by Paenibacillus, and correctly quantify the influence on the by-product metabolite profile of ICGEB2008. Both simulations and experiments showed that metabolic flux is diverted from ethanol to acetate production when an oxidized nitrogen source is utilized.

**Conclusion:**

We have created a predictive model of the central carbon metabolism of *P. polymyxa* ICGEB2008 and could show the presence of the Bifid shunt and explain its role in ICGEB2008. An in-depth study has been performed to understand the metabolic pathways involved in ethanol, 2,3-butanediol and hydrogen production, which can be utilized as a basis for further metabolic engineering efforts to improve the efficiency of biofuel production by this *P. polymyxa* strain.

**Electronic supplementary material:**

The online version of this article (doi:10.1186/s13068-015-0338-4) contains supplementary material, which is available to authorized users.

## Background

The ecological roles of *Paenibacillus polymyxa* are highly diverse and have been described in the literature for various important symbiotic relationships. It was found to be associated with plants where it helps in N_2_ fixation [1] and secreting growth hormones [2], and it was also observed in the gut of insects where it helps the insects to digest their food by secreting various enzymes capable of hydrolyzing organic materials [3]. *P. polymyxa* is a non-pathogenic strain for the production of industrially valuable 2,3-butanediol [4], which is a precursor for various fuels and petrochemical products. So far genome sequences of 14 *P. polymyxa* strains have been deposited at NCBI. However, the genome annotation is far from complete and, to our knowledge, metabolic reconstructions have not been performed for any *Paenibacillus* sp.

The investigation of metabolic pathways allows in-depth insight into the molecular mechanisms of a particular organism. The improved understanding of the architecture of cellular metabolism and the enormous amount of genomic data available today can help draw the entire metabolic map of a cell and redesign it by rational and directed metabolic engineering. The detailed biosynthetic pathways have been established for several model microorganisms such as *Escherichia coli* [5, 6] and *Saccharomyces cerevisiae* [7, 8]. But, despite wide industrial use and availability of the genome sequence for *P. polymyxa*, very little is known about its metabolism [9, 10].

In this report, the metabolic capabilities of *Paenibacillus* sp. ICGEB2008 (referred to as ICGEB2008) [11] have been studied. This strain was isolated from the gut of a cotton bollworm and was shown to produce a number of cellulolytic enzymes [12, 13]. The strain also showed the ability of producing high yields of 2,3-butanediol [14]. In combination, these metabolic capabilities make this strain an interesting candidate for biotechnological purposes, which include the conversion of biomass to combustible fuels or valuable chemicals. To interpret the results obtained from sequence analysis and to obtain new insights into the biochemical capabilities of this strain, we reconstructed a metabolic network model of the carbon metabolism of ICGEB2008 by integrating genomic and biochemical data, resulting in a stoichiometric model connecting 133 metabolites by 158 reactions. Employing flux balance analysis (FBA) [15, 16], we could support the putative annotation of a formate hydrogen lyase and enzymes of the Bifid shunt. The pathways involved specifically in ethanol and 2,3-butanediol production have been studied in detail, generating an understanding that will be useful for engineering the Paenibacillus system for improved biofuel production. We simulated growth on different nitrogen sources and predicted the resulting composition of by-products excreted into the medium. For experimental confirmation, we performed growth experiments, which supported the simulated by-product profiles. In combination, the model and data presented here can serve as a basis for further metabolic engineering and provide an improved insight into the metabolic capabilities of ICGEB2008.

## Results and discussion

### Pathways for fermentative products

**Fig. 1:**
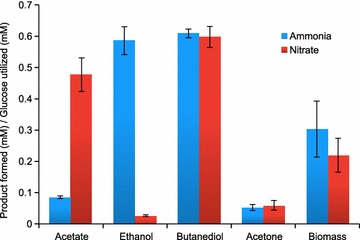
Product profile of *P. polymyxa* ICGEB2008 under anaerobic condition of growth with two different nitrogen sources. The experiments were performed in triplicate by growing the culture in 50 ml medium for 24 h and analyzing the extracellular metabolites via HPLC. The results represent average and standard deviation of data from three biological replicates

Several reports highlight *P. polymyxa* as a non-pathogenic, non-obligatory host for 2,3-butanediol production [4, 17]. Our experiments confirmed this for ICGEB2008 showing a maximal yield of 0.32 g 2,3-butanediol per g of glucose (~0.49 mM 2,3-butanediol/mM glucose) (Fig. 1) [14]. In addition, we also observed ethanol secreted with a yield of 0.18 g per g glucose (~0.7 mM ethanol/mM glucose), besides small amounts of acetic acid, acetone and lactic acid. Sequence analysis confirmed the presence of genes encoding enzymes involved in 2,3-butanediol (*als*, *aldB*, *bdh*), ethanol (*adh*, *ald*) and acetic acid (*pta, ack*) production (Additional file 1: Figure S1, accession number available in Additional file 2). Further, enzymes involved in acetone production were annotated. Interestingly, a gene encoding fructose-6-phosphate phosphoketolase (F6PK) was annotated, which is responsible for a glycolytic bypass pathway called Bifid Shunt. We experimentally validated the functionality of the Bifid shunt by confirming the F6PK activity as 43 nmol/min/mg cellular protein in ICGEB2008 (Additional file 1: Figure S2).

**Fig. 2:**
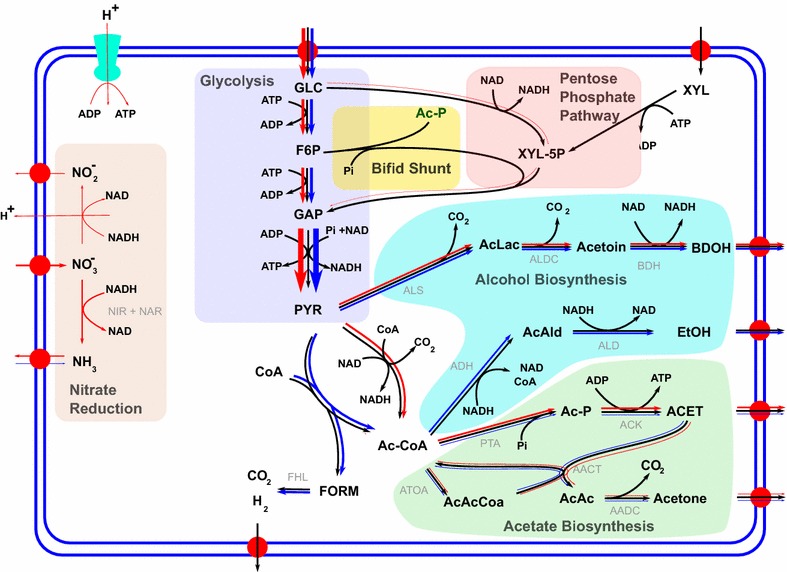
Representation of the predicted flux distributions with nitrate (*red arrows*) and ammonia (*blue arrows*) nutrition. Thickness of *arrows* is proportional to flux values. The two main differences are the use of reactions producing NADH under nitrate nutrition and the employment of FHL as reductant valve during ammonia nutrition. Nitrate reduction is represented by two different processes in the model. Either nitrate is used as final acceptor in the electron transfer chain yielding nitrite, or as source for the NIR + NAR reduction to ammonia

The “Bifid shunt”, or glucose catabolism through the fructose 6-phosphate phosphoketolase (EC 4.1.2.22), generates acetyl phosphate and erythrose 4-phosphate. The final products of the fermentation route are formed by the sequential action of the enzymes: transaldolase (EC 2.2.1.2), transketolase (EC 2.2.1.1.) and xylulose 5-phosphate phosphoketolase (EC 4.1.2.9) which generates glyceraldehyde 3-phosphate, that enters the Embden–Meyerhof–Parnas pathway [18], and acetyl phosphate, which is converted to the final product acetate. Conversion of glucose to acetate through the standard glycolytic pathway yields 4 molecules of ATP and 2 molecules of NADH per molecule glucose (Eq. 1), which also corresponds to the maximal ATP yield (2/3 ATP per carbon, see Table 1). However, the Bifid shunt (Fig. 2) allows to bypass glycolysis and to convert 100 % of the carbons in glucose into acetate (value 1 in Table 1). This pathway results in a lower yield of ATP (2 ATP per glucose), but does not produce reductants (Eq. 2).

**Table 1:** Conversion and production yields on a per carbon basis

Substrate⇒	Cellobiose	Glucose	Glycerol	Xylose
Product⇓				
Acetate	1.0	1.0	1.0 (0.67)	1.0
Acetone	0.75	0.75	0.71 (0.2)	0.75
Butanediol	0.73	0.73	0.83 (0.67)	0.73
Ethanol	0.67	0.67	0.78	0.67
Energy (ATP)	0.75 (0.5)	0.66 (0.42)	0.66 (0.11)	0.66 (0.5)
Biomass	0.72 (0.68)	0.64 (0.59)	0.63 (0.17)	0.64 (0.59)

Energy in ATP refers to the possible hydrolysis of ATP into ADP and Pi per consumed carbon of the given substrate. To quantify the effect of surplus reductants on the results, we performed the simulations without the FHL reaction. The corresponding numbers are given in parentheses

1 $$1\,{\text{Glucose}} \to 2{\text{ Acetate + 4 ATP + 2CO}}_{ 2} {\text{ + 2 NADH }}\left( {\text{Glycolytic route}} \right)$$

2 $$1\, {\text{Glucose}} \to 3 {\text{ Acetate + 2ATP}}\,\left( {\text{Bifid Shunt}} \right)$$

The simultaneous presence of these two pathways introduces a degree of flexibility into metabolism by allowing uncoupling of the production of energy and redox equivalents. As discussed below, this regulatory potential is critically important for anaerobic growth, where electron sinks are not abundant.

### Broad substrate specificity

Due to its capability of producing a number of enzymes hydrolyzing diverse polymeric, oligomeric or dimeric carbohydrates [12, 13], ICGEB2008 is able to grow on a broad spectrum of substrates. This is supported by genome sequence analysis revealing the presence of monosaccharide and disaccharide transporters, allowing utilization of the breakdown products (Table 2). This was validated experimentally by growing ICGEB2008 on different carbon sources under anaerobic conditions (Table 2). Using constraint-based modeling, we calculated maximal carbon and energy yields for various substrates. For a variety of natural carbon sources, the maximal yields of potential products (in carbon per carbon) and ATP (in molecules ATP per carbon) are presented in Table 1. The highest ATP yield per carbon is observed for cellobiose, which is slightly higher than for glucose because of the phosphorylative degradation via cellobiose phosphorylase (CEP) [19].

**Table 2:** Sugar transporters annotated in the genome of *P. polymyxa* ICGEB2008

Sugar	Transporter	Protein ID	Substrate utilized^a^
Glucose	PTS system 2C glucose-specific components	WP_017426741.1, WP_016818550.1	5.0 g/l
Xylose	Xylose ABC transporter 2C permease component	WP_017426799.1, WP_017426800.1, WP_017426801.1, WP_017427837.1, WP_017428351.1	3.78/l
Sucrose	PTS system 2C sucrose-specific component	WP_017426394.1, WP_017426681.1, WP_016820208.1	4.52/l
Maltose	Maltose/maltodextrin ABC transporter 2C permease protein MalF	WP_017427403.1	4.45 g/l
Lactose	Lactose transport system (lactose-binding protein)	WP_017425625.1	4.16 g/l
Cellobiose	PTS system 2C cellobiose-specific component	WP_017428673.1, WP_017428674.1, WP_016819955.1	5.0 g/l
Xylobiose	Xyloside transporter XynT	WP_017427857.1, WP_017426938.1	1.49 g/l
Polysaccharide	Polysaccharide ABC transporter substrate-binding protein, polysaccharide ABC transporter permease	WP_017425534.1, WP_016822415.1	1.18 g/l (Starch)

^a^HPLC measurement for substrate unutilized when ICGEB2008 was grown for 48 h in minimal media containing 5 g/l of respective substrate

### Reconstruction and analysis of a metabolic model

Mathematical models are useful to understand the biochemical capabilities of an organism and to make predictions about the impact of genetic and/or environmental perturbations on metabolic fluxes and growth. Clearly, such understanding supports the development of targeted strategies to improve the yield of the valuable product 2,3-butanediol in biotechnological applications. Therefore, we constructed a stoichiometric model of ICGEB2008 focussing on carbon metabolism. From the genome sequence and the MetaCyc database [20], we assembled a network model containing 133 metabolites and 158 reactions (for details see “Methods”). A representation of the central metabolic routes is given in Additional file 1: Figure S1. To simulate growth, we included in the model reactions describing the conversion of precursors from primary metabolism (such as pyruvate, succinate and ammonia) into the biomass components for DNA/RNA (i.e., single nucleotides), proteins (i.e., connected chains of amino acids), lipids (i.e., glycerolipids) and cell wall (i.e., peptidoglycans). These processes are described as ‘lumped’ reactions, in the sense that they represent a series of biochemical processes instead of single enzymatic reactions. In the following, ‘biomass’ is measured in the unit of incorporated carbon. We confirmed that the model is capable of producing cellular precursors in experimentally determined ratios from minimal medium on various carbon sources (glucose, xylose, cellobiose and glycerol). We used the experimentally observed cellular composition as a constraint to further analyze the model (Table 3).

**Table 3:** Biomass composition for *P. polymyxa* ICGEB2008

Cellular component	Composition (%)
Protein	48.3
DNA/RNA	5.55
Lipids	6.8
Cell wall	20

### Limitations of anaerobic growth

The production of 2,3-butanediol and ethanol was observed majorly under anaerobic conditions (Additional file 1: Figure S3). So, we imposed additional constraints to mimic anaerobic lifestyle in ICGEB2008. The most important constraint is the lack of oxygen as electron acceptor. This poses severe limitations on the overall metabolism, requiring alternative electron sinks. Most importantly, in the absence of oxygen, the strain is not able to produce ATP through oxidative phosphorylation, which imposes a major limitation for anaerobic growth. For example, whereas maximal carbon yields using sugars as substrates are independent of the presence of a redox dissipating mechanism, the biomass yield on glycerol, which is more reduced than sugars, is severely reduced under anaerobic conditions and, therefore, requires an additional oxidation (see Table 1). We investigated the most efficient anaerobic pathways in the model to produce ATP if glucose is the only carbon source. The highest yield of ATP can be obtained with a complete conversion of glucose to acetate, where 4 molecules of ATP can be produced per molecule of glucose. This includes an additional molecule of ATP after fermentation of glucose to pyruvate via phosphate acetyl transferase (PTA) and acetate kinase (ACK). However, only small amounts of acetate are experimentally observed as by-product (Fig. 1). This can readily be explained by the strong pH-dependent growth of ICGEB2008 [14], suggesting that the organism avoids the production of excess acidic compounds. We, therefore, imposed an additional constraint on the model and restricted the export of acidic compounds to a total representing the experimentally observed amounts (0.123 mM/mM glucose based on growth on minimal media). Also, the production of acetone allows for a larger fraction of the carbon to be redirected through acetate synthesis, where additional ATP can be produced. While excretion of acetate would yield even higher ATP amounts, it would simultaneously give rise to higher acid export, which is restricted in the model. Acetone export can circumvent this issue while still allowing the generation of an additional ATP after fermentation of glucose to pyruvate. However, only small amounts of acetone were observed in our experiments. It is likely that the limiting reaction for acetone production is the one catalyzed by acetoacetate CoA transferase (ATOAD), because this enzyme is inhibited by physiological levels of its own product acetone and butanol [21]. The latter is particularly interesting, as 2,3-butanediol could have a similar effect, thus reducing the activity to the observed amounts.

### Formate-hydrogen lyase as a redox valve

**Fig. 3:**
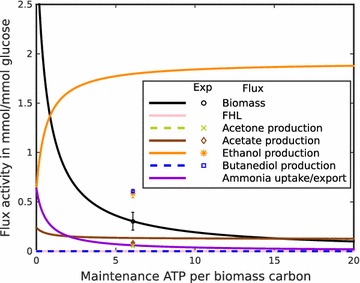
Scan over a range of maintenance ATP required per biomass carbon for the model without formate hydrogen lyase (FHL) activity. One unit of biomass refers to one carbon of newly produced *P. polymyxa*. *Markers* show the experimental values for biomass, ethanol and 2,3-butanediol production

ATP is required for both growth-related and maintenance processes. It is relatively easy to estimate growth-related ATP requirement, but the ATP requirement for non-growth-related processes is far more difficult to assess. One possibility is to systematically vary the flux through an ATP consuming (ATPase) reaction, which mimics the additional ATP maintenance demand, and predict growth and by-product formation and comparing the predicted values with experimentally determined quantities. In Fig. 3, the result of such an ATP requirement scan is depicted. Best agreement with experimentally observed growth is found for an ATPase flux of 6.48 ATP/biomass carbon (Fig. 3), with ethanol as the only predicted by-product. This by-product profile is clearly not in agreement with the experimentally observed mixture of various substances. The model predicts a high ethanol production, mainly because it utilizes ethanol excretion as an efficient way to remove excess reductants from the system. To resolve the discrepancy between model and experiment, we hypothesized the presence of an alternative mode of reductant dissipation. To find the alternate pathway, we analyzed the genome sequence of ICGEB2008 and found a gene coding for a formate-hydrogen lyase (FHL), which utilizes NADH and produces hydrogen. This hydrogenase uses protons as final electron acceptors and can act as a mode to dissipate excess NADH. The hypothesis was supported by hydrogen production in other *Paenibacillus* strains [22], which we could also confirm for ICGEB2008 by GC analysis (Additional file 1: Figure S4).

**Fig. 4:**
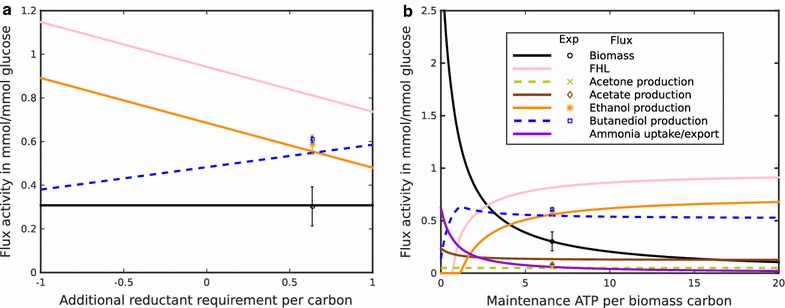
**a** Scan over the strength of an additional electron sink to identify the amount of additional reduction required to predict the experimentally observed by-product formation. Predicted ratio of ethanol/butanediol shifts towards 2,3-butanediol in response to oxidized medium. *Positive values* indicate additional flux through the DEHOG reaction and thus either an increased demand of reductant or a more reduced biomass. *Markers* show the experimental values for biomass, ethanol and 2,3-butanediol production. The simulated outputs fit well to these when using FHL and an adjusted redox demand of +0.7/biomass carbon (see text). **b** Scan over a range of maintenance ATP required per biomass carbon as in Fig. 3, but with FHL and an additional reductant requirement of +0.7/biomass carbon, as determined by Fig. 4a)

Incorporation of this enzyme into the model improved the prediction of by-products considerably (Additional file 1: Figure S5). However, the production of ethanol was still slightly overestimated and the production of 2,3-butanediol slightly underestimated. Thus, the model predicted a more reduced total by-product combination than experimentally observed. To quantify the discrepancy between the observed and predicted requirement of reductants, we included a reductant removing reaction in the model and systematically varied the corresponding flux (Fig. 4a) in analogy to the method described above to estimate maintenance ATP requirements. This analysis revealed a best fit to the observed growth and by-product ratios for an additional consumption of reductant of about 0.7 NADH per biomass carbon (Fig. 4b). To identify the additional electron sink, which could explain the origin of this discrepancy, we analyzed the model prediction for the formation of CO_2_, a byproduct with extreme oxidation state, which was not experimentally measured. The predicted value of CO_2_ formation under the assumption of biomass maximization was 1.96 mM/mM glucose. To study whether this value was potentially overestimated by the model, we systematically fixed the CO_2_ formation to values between 1.5 and 2 mM/mM glucose (Additional file 1: Figure S6). Apparently, with lower CO_2_ production the by-products become less reduced. The best fit to the experimentally observed values was obtained for a value of 1.83 mM CO_2_/mM glucose. This fit is of the same quality as that obtained by assuming an additional electron sink (Fig. 4b). We, therefore, conclude that the discrepancy between model prediction and observed by-product formation (Additional file 1: Figure S5) results from an overestimation of CO_2_ production and that this overestimation can be corrected by assuming an additional electron sink.

### Importance of N-source and altering the redox balance

**Fig. 5:**
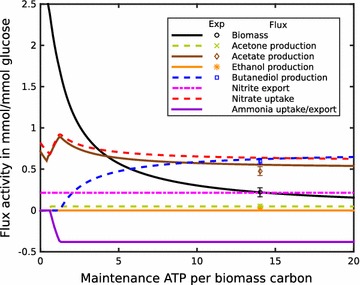
ATP requirement scan with nitrate as nitrogen source. The model predicted ammonium production, which was experimentally confirmed for growth of *P. polymyxa* ICGEB2008 on nitrate. Constraining the total ammonium production in the model to experimentally observed values, the model predicts a decrease in ethanol production to zero and an increase in acetate formation, which is in good qualitative agreement with experimental data. The simulations suggest that the maintenance ATP requirement per biomass almost doubles on nitrate when compared to ammonium nutrition

Given the constraints on anaerobic metabolism by the unavailability of molecular oxygen for the dissipation of reductants, it appears plausible that the redox state of the nutrients will influence by-product metabolite production of ICGEB2008. Since nitrate can be used as a terminal electron acceptor [23], we investigated the effect of supplying nitrate instead of ammonium as nitrogen source. It is expected that under growth on nitrate, reductants are no longer in excess, but rather required for nitrate reduction. The model consequently predicted a strong increase in acetate production and a decrease in ethanol production (Fig. 5). To test this prediction experimentally, we grew ICGEB2008 in a medium in which ammonia was replaced by nitrate and found that the model correctly predicted that 2,3-butanediol and acetate are the only major expected by-products (Fig. 1). The model further suggested that nitrate is used as final electron acceptor and is reduced to ammonia, which is exported. This hypothesis was supported by the genome analysis, which revealed two corresponding nitrate reductase gene clusters in the genome of ICGEB2008 (Additional file 1: Figure S7). For experimental confirmation, we measured the ammonium content in the extracellular medium and found that after 48 h approximately a third of the initially applied nitrate had been reduced to ammonia (Additional file 1: Figure S8). We further observed the presence of nitrite in the final solution and adapted the model accordingly. Constraining nitrite and ammonia export to the experimentally observed values resulted in a predicted by-product composition closely reflecting the experimentally observed values (Fig. 5). The calculated flux distributions for the best fits for nitrate and ammonium nutrition are depicted in Fig. 2. The main differences arise from a flux through reductant producing pathways under nitrate nutrition, such as the use of pyruvate dehydrogenase (PDH) instead of pyruvate-formate lyase (PFL). An additional effect is the inactivation of the Bifid shunt. The availability of nitrate as terminal electron acceptor allows removing excess reductants and, therefore, the standard glycolytic pathway is operable. Thus, in contrast to growth on ammonium, the “reductant free” ATP that can be regenerated by the Bifid shunt is no longer necessary.

## Conclusions

The diverse metabolic capabilities of *Paenibacillus polymyxa*, ranging from the degradation of diverse organic compounds in the gut of insects to the production of industrially relevant biomolecules like 2,3-butanediol, make this organism a particularly interesting candidate for a detailed investigation of its metabolic potential. They also highlight that *P. polymyxa* is of considerable interest for potential biotechnological applications. Here, we demonstrated the capability of the ICGEB2008 strain to produce biofuels, in particular ethanol and 2,3-butanediol. To understand the metabolic pathways operative in ICGEB2008 and to develop strategies to stimulate production of biofuels in this strain, we have built a metabolic model and applied flux balance analysis (FBA) to explain and predict the by-product formation under different growth conditions. Our model successfully predicted the influence of the redox balance in ICGEB2008. Using a combinatorial experimental and modeling approach, we have shown the influence of the redox state of the nitrogen source on by-product yields and composition. We predicted a diversion of metabolic flux from ethanol to acetate in more oxidizing environments, which we experimentally confirmed by growing ICGEB2008 on nitrate. The model further supported the existence of a formate hydrogen lyase as redox valve. By the detection of hydrogen in the overhead, we could further support this finding experimentally. This finding could serve as a starting point for further development of the organism as a potential hydrogen producer as an additional useable by-product. We have also elucidated the presence of the Bifid shunt in the bacterium as a bypass for the glycolytic pathway, which is active under anaerobic growth without the presence of an alternative electron acceptor such as nitrate.

In summary, we have developed a metabolic model for the industrially relevant strain, *Paenibacillus polymyxa* ICGEB2008 and investigated the metabolic pathways operative in this strain. We have demonstrated the potential of metabolic modeling to simulate the capabilities of ICGEB2008 and elucidated the Bifid shunt in *P. polymyxa* ICGEB2008. In addition, we have shown that the redox state of the nitrogen source is critical for the by-product profile. The present study can serve as a basis for further metabolic engineering efforts to improve the efficiency of biofuel production by this *P. polymyxa* strain.

## Methods

### Culture media and cultivation conditions

For biomass composition and product analysis, ICGEB2008 was grown in minimal medium [24] containing 5 g/l glucose. The growth in nitrate medium was obtained by replacing ammonium chloride with an equimolar quantity of sodium nitrate. The effect of acetate on ICGEB2008 metabolism was studied by adding the specified amount of acetate after OD_600_ reached 0.8 and the culture was grown further for 48 h. The products secreted were quantified using HPLC. Substrate specificity was established by growing ICGEB2008 anaerobically in 125 ml serum bottles containing media with 2.5 g/l of different carbon sources. The carbon source utilization was estimated using HPLC. The values obtained for cell biomass, substrate, utilization and product synthesis were used for calculation of biomass and product yields (mmol/mmol substrate). For calculating biomass yield, a molecular formula CH_1.9_O_0.5_N_0.2_ of cells was used with an average molecular weight of 24.7 [25].

#### Cell composition analysis

Different components of cells were analyzed for their composition [26, 27]. Cell wall was extracted by repeated washing of a known mass of ground lyophilized tissue with a mixture of phenol, acetic acid, and water in the ratio 2:1:2 [26]. The remaining insoluble material was washed with distilled water, freeze dried, and weighed as cell wall component. Lipids were extracted from a known mass of ground-lyophilized tissue using hexane and isopropanol according to an established protocol [27]. Solvent was removed by gentle heating, and residues were weighed as lipid component. Soluble protein extracted with phosphate-buffered saline was quantified against BSA standard using the BCA protein assay kit (Bio-Rad). Nucleic acids were extracted from lyophilized tissue using standard methods (i.e., for RNA, TRIzol extraction followed by DNase treatment; for DNA, phenol/chloroform/isoamyl alcohol extraction followed by RNase treatment) and quantified spectrophotometrically.

### Genome annotation and subsystem analysis

The initial annotation of coding sequences of ICGEB2008 was achieved using the automated server RAST (http://rast.nmpdr.org/) [28], which is available at NCBI website with Reference Sequence No. NZ_AMQU00000000.1. While constructing the metabolic pathways, the missing link in the network was identified by Reverse Blast Hit (RBH) strategy with BLAST threshold at 1e^−05^ and their annotations have been provided in the Additional file 2. Gene clusters were analyzed using img/er server (https://img.jgi.doe.gov).

### Analytical assays

Ammonium ion production was estimated as follows. To 1.5 ml culture supernatant, 50 μl manganous salt solution, 1 ml alkaline phenol reagent and 0.5 ml hypochlorite solution were added. The reaction mix was boiled for 5 min and color development was monitored at 625 nm [29]. Residual nitrate estimation was done using a modification of the method described by Middleton [30]. To 0.5 ml of culture filtrate, 5 ml of 0.55 % Ca(CH_3_COO)_2_·H_2_0 in 4 % ammonia, 0.1 ml of 1 % MnSO_4_·4H_2_0 in 5 % acetic acid, and about 0.1 g of finely powdered zinc were added. This mixture was shaken vigorously for 1 min and filtered; 2 ml of the filtrate was placed on ice and 0.5 ml of 1 % sulfanilamide in 5 N HCl was added. The sample was incubated on ice for 15 min followed by the addition of 0.5 ml of 0.02 % *N*-(1-naphthyl)-ethylenediamine solution and incubation at room temperature for 30 min. After incubation, 2 ml of water was added and absorbance was measured at 540 nm in spectrophotometer.

Fructose-6-phosphate phosphoketolase was assayed based on Tannock’s protocol [31] as follows. The cells were harvested by centrifugation after 24-h cultivation in Scheper’s minimal medium. The bacterial cells were washed using 10 ml of 0.05 M phosphate buffer and finally suspended in 1 ml of phosphate buffer containing 3 mg lysozyme. The cells were then lysed by sonication used for the assay. The cell lysate (100 μl), sodium fluoride-iodoacetic acid solution (24 μl of 6 mg/ml) and fructose-6-phosphate (24 μl of 12 mg/ml) were added to the test wells and the reaction was incubated at 37 °C for 30 min. The fructose-6-phosphate was not added in the negative control well. The reaction was stopped by adding 150 μl of hydroxylamine solution, 100 μl of trichloroacetic acid solution and 100 μl of 4 M hydrochloric acid. A reddish-violet color was obtained after addition of 100 μl of ferric chloride solution indicating fructose-6-phosphate phosphoketolase activity, which was estimated spectrophotometrically at 505 nm.

Hydrogen was estimated by growing **c**ells anaerobically in minimal media in the sealed serum bottle for 36 h and headspace gases were analyzed by GC (Carboxen-1010 Plot column in Perkin Elmer’s Clarus 500GC) for hydrogen estimation.

### Model construction and curation

The metabolic model of ICGEB2008 [11] was reconstructed based on an initial annotation of the central carbon metabolism by RAST [28] and protein name and EC number matching in MetaCyc. To reduce complexity, batch reactions for amino acid, nucleotide and lipid biosynthesis were introduced based on MetaCyc Pathways. ScrumPy [32] was used as modeling tool and to perform flux balance analysis with a dual objective of biomass optimization followed by flux minimization. The solver employed was CPLEX 12.6 with an interface for ScrumPy. To obtain a comprehensive and organism specific network, we extracted all reactions catalyzed by enzymes from both the central carbon metabolism and fermentation subcategories of RAST’s carbohydrate metabolism group. For these enzymes, we extracted the EC numbers and retrieved the corresponding reactions from MetaCyc using ScrumPy as modeling tool. The retrieved reactions were manually filtered as many retrieved EC numbers link to unspecific reactions (e.g., aldehyde dehydrogenase). In MetaCyc these EC numbers can match very specialized reactions which do not have their own EC number and are only present in specialized organisms. In addition, we removed reactions which produce compounds that were not observed in *Paenibacillus* and were only present in single reactions.

We introduced batch reactions for biosynthesis of the 20 proteinogenic amino acids and all nucleotides (four ribonucleic acids and four deoxyribonucleic acids). Finally, we added two batch reactions for lipid biosynthesis—one forming palmitic acid and another to convert three palmitic acids and glycerol-phosphate into one lipid. We chose this simplification over the actual lipid metabolism because the general requirements for energy and reductant are similar enough for our study between the different lipids. To create these batch reactions, the biosynthesis pathways from MetaCyc were combined into one reaction by adding up all substrates and products.

The current model is restricted to anaerobic conditions and, therefore, works under a strict redox balance. However, we have included a batch electron transfer chain using nitrate as substrate. This reaction mainly converts ADP, Pi, nitrate and NADH to NAD^+^, water, nitrite and ATP.

### Network reconstruction and modeling

Metabolic models allow us to investigate the metabolism of the modelled organisms. A robust method often used in this kind of studies is flux balance analysis (FBA) [15]. The huge advantage of FBA is that it allows an investigation of the desired organism without prior knowledge of enzyme kinetics. FBA is solely based on reaction stoichiometries and assumes an internal steady state for all metabolites, while allowing the uptake and production of compounds. Since kinetic information is most often limiting, this approach has proven useful for many larger scale networks but can also be applied readily to small sub-networks covering the most important constraints of the system.

### Modeling techniques

The general formulation of the linear problem solved in FBA is:

3 $$\begin{aligned} &{\text{Optimize }}Z \hfill \\ &s.t. \hfill \\ &N \, \cdot \, \vec{v} \, = \, 0 \hfill \\ &a_{j} \le v_{j} \le b_{j} \hfill \\ \end{aligned}$$

where *N* is the stoichiometric matrix of all reactions and *v* is the flux vector representing the reaction fluxes. *a*_*j*_ and *b*_*j*_ are the lower and upper bounds for each flux *v*_*j*_ in *v*. For irreversible reactions, *a*_*j*_ is set to 0, while as default no other bounds are applied. We performed several scans to investigate maintenance energy requirements and redox state. These scans are based on the common FBA formulation. Scanning is then performed by adding a constraint (*a*_*j*_ = *b*_*j*_) in Eq. (3) or by adding a sum constraint to address questions like ATP per biomass carbon. For ATP per biomass carbon, the constraint would look like:

4 $$c \cdot v_{\text{Biomass}} {-} \, v_{{{\text{ATP}}\,{\text{consumption}}}} = \, 0$$

where *c* would be the amount of ATP per produced biomass. To reduce the influence of possible alternate optima, each optimal solution was then flux minimized. This is achieved by splitting all reactions into forward and backward reactions, and minimizing the total flux in the network. To retain the detected optima (e.g., maximal biomass), the biomass flux was fixed at the optimal value during minimization. All modeling was performed using ScrumPy. CPLEX was used as solver for FBA problems.

The model and the source code for model analysis is available at https://github.com/QTB-HHU/Paenibacillus. A list of all the reactions and accession numbers for all the enzymes carrying out these reactions  is provided as Additional file 2.

## Additional files

**Additional file 1:** Additional figures and tables.
**Additional file 2:** List of all reactions contained in the network and the corresponding enzyme accession numbers.

## Abbreviations

FBA: flux balance analysis
RAST: rapid annotation using subsystem technology
BDO: 2,3-butanediol
PTA: phosphotransacetylase
ACK: acetate kinase
ALDC: acetolactate decarboxylase
ATOAD: acetoacetate CoA transferase
AADC: acetoacetate decarboxylase
ALS: acetolactate synthase
ADH: acetaldehyde dehydrogenase
ALD: aldehyde/alcohol dehydrogenase
FHL: formate-hydrogen lyase
BDH: 2,3-butanediol dehydrogenase
NAR: nitrate reductase
NIR: nitrite reductase
ATOB: acetyl-Coa acetyltransferase
