# Supplementary material for: Insight into metabolic pathways of the potential biofuel producer, Paenibacillus polymyxa ICGEB2008
Source: Biotechnol Biofuels. 2015 Sep 25;8:159. doi: 10.1186/s13068-015-0338-4 (PMC4583153; doi:10.1186/s13068-015-0338-4)
Supplement: Supplementary file 1 — Additional file 1: Additional figures and tables. [file 13068_2015_338_MOESM1_ESM.doc]

**Additional Figures**

**Figure S1.** Schematic representation of the central carbon metabolism of *P. polymyxa* ICGEB2008. Metabolite colors indicate their involvement in the following pathways: Yellow: Glycolysis; Purple: TCA; Blue: Pentose phosphate pathway; Green: Byproduct formation pathways. The Bifid shunt is depicted in orange; The succinate dehydrogenase (Red Arrow) is restricted under Ammonia conditions. Enzyme Abbreviations: ACK -   Acetate kinase; SDH  -  Succinate dehydrogenase; ALDC -   Acetolactate decarboxylase; AACT  -  Acetoacetate CoA transferase; CEP  -  Cellobiose phosphorylase; AADC  -  Acetoacetate ; decarboxylase; F6PK  -  Fructose-6-phosphate phosphoketolase; ALS  -  Acetolactate synthase; PTA  -  Phosphate acetyl transferase; ADH  -  Acetaldehyde dehydrogenase; ALD  -  Aldehyde/alcohol dehydrogenase; FHL  -  Formate-hydrogen lyase; BDH  -  2,3-butanediol dehydrogenase; ATOB  -  Acetyl-Coa Acetyltransferase


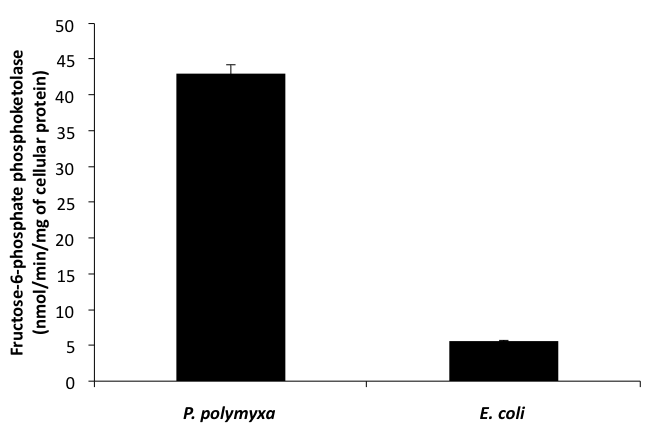


**Figure S2.** Activity of fructose-6-phosphate phosphoketolase, a key enzyme of Bifid shunt, in *P. polymyxa* ICGEB2008 as compared to *E. coli.*


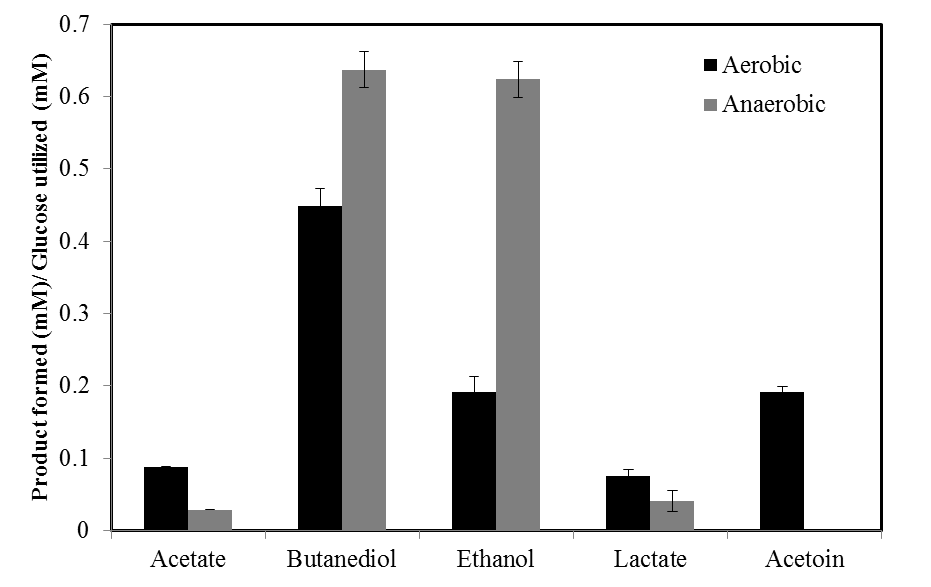


**Figure S3.** Product profile of *P. polymyxa* ICGEB2008 under aerobic and anaerobic condition*.*The experiments were performed in triplicate by growing the culture in 50 ml medium for 24 hr and analyzing the extracellular metabolites via HPLC.

Hydrogen

**Supplementary Fig. 3**. GC analysis of exhaust gas of *P. polymyxa* ICGEB2008 showing hydrogen production under anaerobic condition.

**Figure S4**. GC analysis of exhaust gas of *P. polymyxa* ICGEB2008 showing hydrogen production under anaerobic condition.

**Figure S5** Scan over a range of maintenance ATP required per biomass carbon for the model with formate hydrogen lyase (FHL) activity, but without additional consumption of reductant. One unit of biomass refers to one carbon of newly produced *P. polymyxa*. Markers show the experimental values for biomass, ethanol and 2,3-butanediol production.

**Figure S6** Predicted byproduct formation for the model with FHL activity but without additional reductant requirement, where the carbon dioxide production is systematically fixed between values of 1.5 and 2mM/mM glucose. Markers show the experimental values for biomass, ethanol and 2,3-butanediol production. A best fit to the experimental values is achieved for a fixed carbon dioxide production of 1.83 mM/mM glucose.

Nitrate reductase gene cluster (nucleotide position - Scaffold 8: 256993-274102)

*
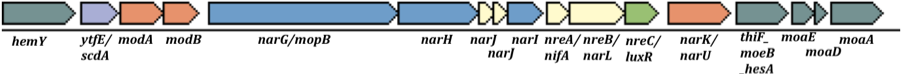
*

Nitrite reductase gene cluster (nucleotide position- Scaffold 19: 59068-70129)


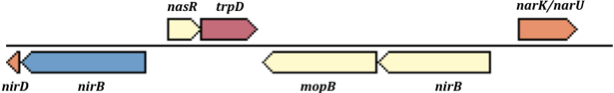


**Figure** S**7**. Nitrate/nitrite reductase gene clusters present in the genome of *Paenibacillus polymyxa* ICGEB2008. Gene abbreviations: *hemY* - protoporphyrinogen oxidase; *ytfE/scdA* - iron-sulfur cluster repair di-iron protein (protect from NO damage); *modA*- molybdenum ABC transporter substrate-binding protein; *modB­*- molybdenum ABC transporter permease; HP – hypothetical protein; *narG/mopB*- respiratory nitrate reductase A (NarGHI), alpha chain (NarG)/molybdopterin oxidoreductase; *narH*- respiratory nitrate reductase, beta subunit; *narJ*- nitrate reductase delta subunit; *narI*- nitrate reductase gamma subunit; *nifA/nreA*- DNA-binding regulatory protein (GAF/*nifA*/*nreA*/*fhl* activator/control of N_2_ reduction); *nreB/narL-* histidine kinase-, DNA gyrase B-, and HSP90-like ATPase; *nreC/luxR*- response regulator, *luxR* family; *nark/narU*- Major Facilitator Superfamily/nitrate/nitrite transporter; *thiF-moeB-hesA*- dinucleotide-utilizing enzymes involved in molybdopterin and thiamine biosynthesis family 2; *moaE*- molybdopterin synthase subunit; *moaD*- molybdopterin synthase subunit; *moaA­-* molybdenum cofactor biosynthesis protein A; *nirD*- nitrite reductase [NAD(P)H], small subunit; *nirB*- nitrite reductase [NAD(P)H], large subunit; *nasR*- response regulator with putative antiterminator output domain (ANTAR); *trpD*- anthranilate phosphoribosyltransferase; *mopB*- nitrate reductases, NapA, NasA, and NarB catalyze the reduction of nitrate to nitrite (MopB supfamily); *narK/narU­*- nitrate/nitrite transporter, Major Facilitator Super Family. Colour coding is based on COG clustering.

**
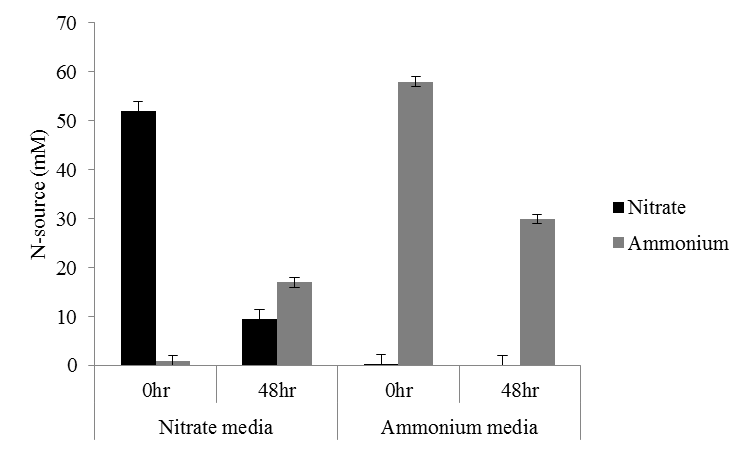
**

Figure S8. The presence of ammonium ion in the culture media containing nitrate as the sole nitrogen source.

**Additional information to Model**

**List of coding sequences annotated using RAST (not found in NCBI)**

**Butyryl CoA dehydrogenase -** [fig|6666666.25310.peg.1020](http://rast.nmpdr.org/seedviewer.cgi?page=Annotation&feature=fig|6666666.25310.peg.1020)

MSSGSAIQQLAGREAFRSFAERYIVHEADRWDSEEAIPQSIIQQMGAEGYLGAVISKEFG

GLELDMKSLGALSEEMGRACSSVRSLLTVHGMASIAVERFGTAEQRQKWLPLLASGQTIG

AFGLSEAGAGTDTKAITTTATITEEGYVLEGGKKWITFGQIADLFLIFAKLDGEPTAFLV

PRTTPGLSIDHLKGIFGTTASMIAELRMENCLIPHEAILGSKGLGVPYIAMSCLDYGRYT

IAWGCVGILQACLDACLSYTSKRETFGTLLKNQQLIQKMITEMTVNTKAARLLCEDAGRL

KDEGDSQGLLATWAAKYFASISATKAANEAVQIHGANGCSRDYPVQRYLRDAKVMEIIEG

TTQMHELVISSDAYSLSDF

**Dihydroxyacetone Kinase -** [fig|6666666.25310.peg.4817](http://rast.nmpdr.org/seedviewer.cgi?page=Annotation&feature=fig|6666666.25310.peg.4817)

MKKVINRTENLVLEMCSGIALAHPELEFLPKYKVLKKKELNTEKVTLISGGGSGHEPAHA

GFVGKGMLDAAVCGDVFASPSQIQVYQAIRATAGKKGTLLIIKNYSGDIMNFRNGAHLAA

EDGLEVDYVKIEDDIAVEDSLYTVGRRGVAGTVLVHKIAGAAAEEGRSLAEVKAVAEKAA

ANVRSIGFALTSCTVPAKGSPTFELGPDEIEYGVGIHGEPGIRREKLTDADTLARRMVTD

LLRDMQIENGFSGEIALLINGFGGTPLQELYLFNYAVTRELAAKGIKINRSFVNNYMTSI

DMAGISVSIMKLDDELQTLLSHESNTPAFKVSGPADQVVFTDLSTVADENRQVSFEVETA

PAFAVIEDNKITLNNLIYLVDKMSEVIIKNEVPFCDLDSHAGDGDFGMSVAKGFRQLKRE

WSDILHQDSLTMGSFLHSCSLVIMEYCGGASGPIWGSAFRAAARSAGERSELTIAEFADM

MQAAVQGIQSTGERSFGRGAVVGDKTLIDALVPCADSWVESAKSNVDVKEAFVRGAQAAV

LGAKKTENIVARMGRAGAVGERSLGYPDAGAYALGVIFTELAECLN
